# Supplementary material for: Immune Responses and Transcriptomic Analysis of Nilaparvata lugens against Metarhizium anisopliae YTTR Mediated by Rice Ragged Stunt Virus
Source: Plants (Basel). 2023 Jan 11;12(2):345. doi: 10.3390/plants12020345 (PMC9865581; doi:10.3390/plants12020345)
Supplement: Supplementary file 1 [file plants-12-00345-s001.zip › plants-2122077-supplementary-new-down.pdf]

plants MDPI

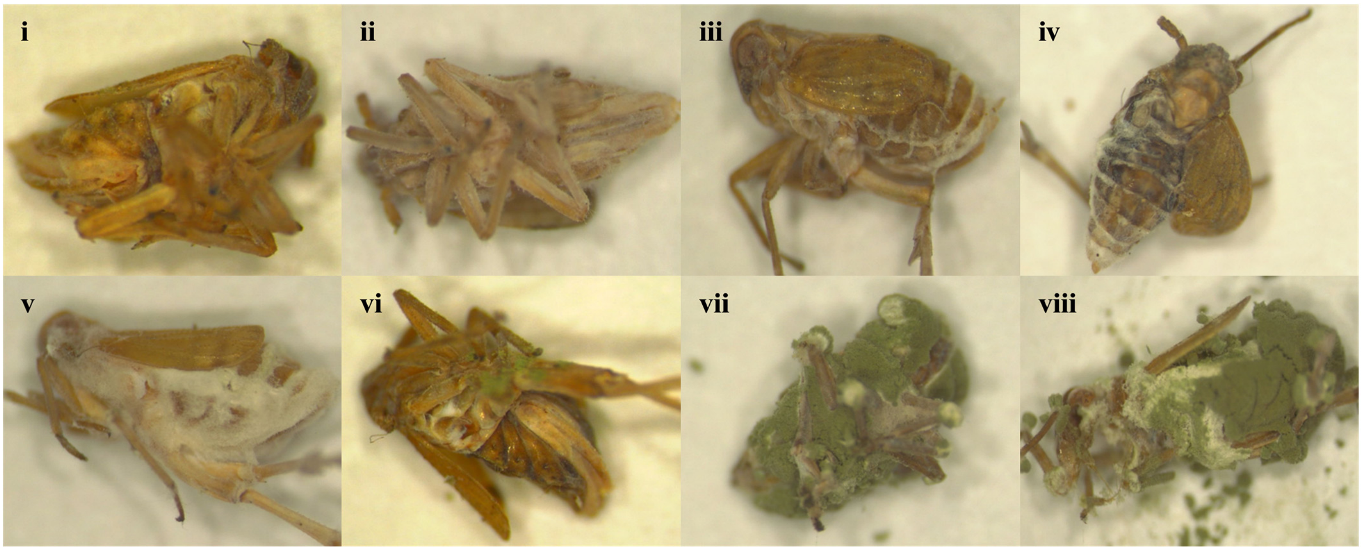

Figure S1. The disease symptoms of *Metarhizium anisopliae* YTTR-infected BPH (mycosis). i means the initial post-infection cadaveric symptoms of BPH. ii-v means the procedure of hyphae germination. vi-viii means the procedure of sporulation.

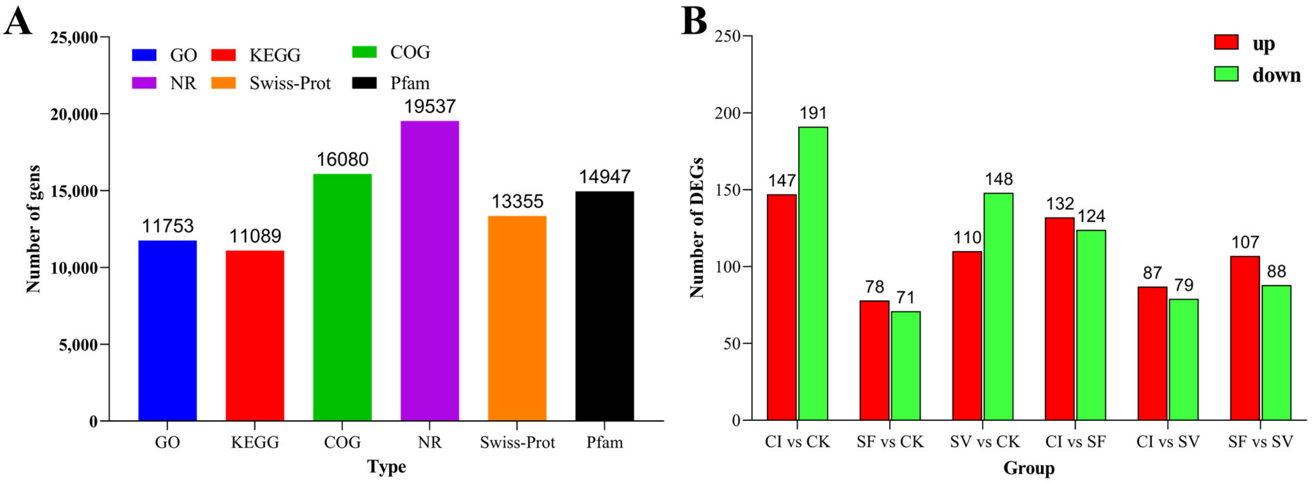

Figure S2. The number of annotated genes in major databases (**A**) and DEGs in different treatment groups (**B**). RRSV-free BPH were treated with Tween-80 (CK) or *Metarhizium anisopliae* YTTR (SF) and RRSV-carrying BPH were also treated with Tween-80 (SV) or *M. anisopliae* YTTR (CI), respectively. The expression of DEGs among the four treatment groups (CK, SF, SV, and CI) were compared by any two treatment groups. CI vs SF, CI vs CK, CI vs SV, SV vs CK, SF vs CK, and SF vs SV were defined

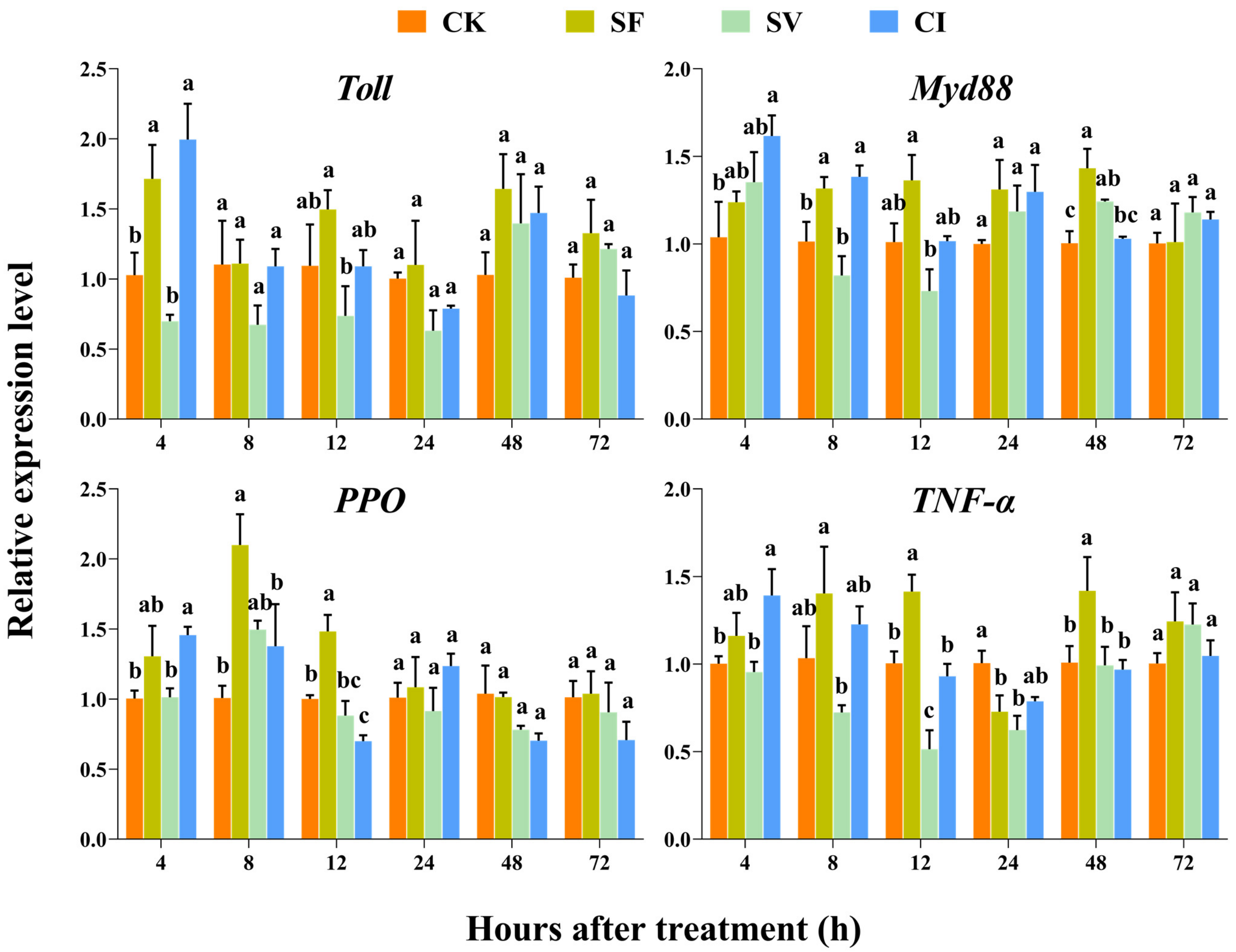

Figure S3. RRSV-mediated effects on the expression of immune genes of BPH encountering Metarhizium anisopliae YTTR infection. RRSV-free BPH were treated with Tween-80 (CK) or M. anisopliae YTTR (SF) and RRSV-carrying BPH were also treated with Tween-80 (SV) or M. anisopliae YTTR (CI). After 4, 8, 12, 24, 48, and 72 h of treatment, these genes' expression were measured by qRT-PCR. Data are mean ± SEM (n = 10), and different lowercase letters indicate significant differences in gene expression between treatments within the same treatment time ($p < 0.05$, ANOVA, Duncan's test).

Table S1. RNA Sequencing data statistics and comparison results with reference genome.

Sample	Raw Reads	Clean Reads	Q30 (%)	GC Content (%)	Total Mapped	Uniquely Mapped	SRA Accession
CK1	60.9G	58.4G	93.00	46.69	45.5G(77.91%)	38.2G(65.48%)	SRR21672257
CK2	58.1G	56.2G	93.29	47.66	44.4G(79.07%)	38.4G(68.35%)	SRR21672380
CK3	43.0G	41.4G	93.41	47.68	32.9G(79.34%)	28.5G(68.78%)	SRR21673162
SF1	44.3G	42.8G	93.11	47.09	33.7G(78.74%)	28.8G(67.35%)	SRR21673296
SF2	48.6G	47.2G	93.35	48.33	36.7G(77.78%)	31.0G(65.81%)	SRR21674161
SF3	54.3G	52.6G	92.72	47.28	41.9G(79.73%)	36.3G(68.97%)	SRR21675536
SV1	43.9G	42.8G	93.56	47.23	33.9G(79.26%)	28.6G(66.90%)	SRR21720405
SV2	58.9G	57.4G	93.37	47.72	45.3G(78.85%)	39.0G(67.95%)	SRR21675632
SV3	49.0G	48.0G	93.31	47.86	37.9G(78.87%)	32.6G(67.95%)	SRR21675714
CI1	46.6G	45.7G	93.24	47.72	35.9G(78.63%)	30.7G(67.35%)	SRR21679236
CI2	47.4G	46.7G	93.72	47.57	36.7G(78.69%)	31.3G(67.11%)	SRR21679328
CI3	53.3G	51.7G	93.09	47.56	40.7G(78.8%)	34.6G(67.05%)	SRR21679458

RRSV-free BPH were treated with Tween-80 (CK) or *Metarhizium anisopliae* YTTR (SF) and RRSV-carrying BPH were also treated with Tween-80 (SV) or *M. anisopliae* YTTR (CI).

Table S2. Analysis of KEGG annotation of DEGs expressed only in the comparison of CI vs CK alone.

Category	Pathway ID	Description	Gene Numbers
Metabolism			
Amino acid metabolism	map00310	Lysine degradation	2
Lipid metabolism	map00061	Fatty acid biosynthesis	1
Lipid metabolism	map00140	Steroid hormone biosynthesis	1
Amino acid metabolism	map00260	Glycine, serine and threonine metabolism	2
Carbohydrate metabolism	map00053	Ascorbate and aldarate metabolism	2
Lipid metabolism	map00564	Glycerophospholipid metabolism	1
Xenobiotics biodegradation and metabolism	map00983	Drug metabolism—other enzymes	1
Metabolism of terpenoids and polyketides	map00981	Insect hormone biosynthesis	1
Lipid metabolism	map00561	Glycerolipid metabolism	3
Carbohydrate metabolism	map00052	Galactose metabolism	1
Metabolism of cofactors and vitamins	map00760	Nicotinate and nicotinamide metabolism	1
Glycan biosynthesis and metabolism	map00601	Glycosphingolipid biosynthesis—lacto and neolacto series	1
Lipid metabolism	map00591	Linoleic acid metabolism	1
Glycan biosynthesis and metabolism	map00510	N-Glycan biosynthesis	3
Glycan biosynthesis and metabolism	map00513	Various types of N-glycan biosynthesis	3
Glycan biosynthesis and metabolism	map00514	Other types of O-glycan biosynthesis	1

Glycan biosynthesis and metabolism	map00515	Mannose type O-glycan biosynthesis	2
Lipid metabolism	map00100	Steroid biosynthesis	1
Lipid metabolism	map00590	Arachidonic acid metabolism	1
Metabolism of terpenoids and polyketides	map00900	Terpenoid backbone biosynthesis	1
Amino acid metabolism	map00270	Cysteine and methionine metabolism	2
Energy metabolism	map00190	Oxidative phosphorylation	1
Amino acid metabolism	map00220	Arginine biosynthesis	2
Carbohydrate metabolism	map00030	Pentose phosphate pathway	2
Metabolism of cofactors and vitamins	map00830	Retinol metabolism	2
Nucleotide metabolism	map00230	Purine metabolism	4
Biosynthesis of other secondary metabolites	map00232	Caffeine metabolism	1
Amino acid metabolism	map00330	Arginine and proline metabolism	2
Glycan biosynthesis and metabolism	map00533	Glycosaminoglycan biosynthesis—keratan sulfate	1
Lipid metabolism	map01040	Biosynthesis of unsaturated fatty acids	2
Carbohydrate metabolism	map00520	Amino sugar and nucleotide sugar metabolism	1
Nucleotide metabolism	map00240	Pyrimidine metabolism	1
	map04120	Ubiquitin mediated proteolysis	2
Folding, sorting and degradation	map03018	RNA degradation	1
	map04141	Protein processing in endoplasmic reticulum	3
	map03013	RNA transport	2
Translation	map03015	mRNA surveillance pathway	1
	map03010	Ribosome	3
Transcription	map03022	Basal transcription factors	1
Environmental Information Processing			
	map04024	cAMP signaling pathway	2
	map04390	Hippo signaling pathway	1
	map04391	Hippo signaling pathway—fly	2
	map04014	Ras signaling pathway	1
	map04010	MAPK signaling pathway	2
	map04013	MAPK signaling pathway—fly	1
	map04012	ErbB signaling pathway	1
Signal transduction	map04310	Wnt signaling pathway	3
	map04371	Apelin signaling pathway	2
	map04341	Hedgehog signaling pathway—fly	1
	map04020	Calcium signaling pathway	2
	map04022	cGMP-PKG signaling pathway	2
	map04668	TNF signaling pathway	1
	map04068	FoxO signaling pathway	1
	map04064	NF-kappa B signaling pathway	1
	map04150	mTOR signaling pathway	1

	map04152	AMPK signaling pathway	3
	map04071	Sphingolipid signaling pathway	1
Signaling molecules and interaction	map04514	Cell adhesion molecules (CAMs)	1
	map04080	Neuroactive ligand-receptor interaction	1
Membrane transport	map02010	ABC transporters	1
Cellular Processes			
Cellular community—eukaryotes	map04510	Focal adhesion	1
	map04530	Tight junction	1
	map04550	Signaling pathways regulating pluripotency of stem cells	1
Transport and catabolism	map04137	Mitophagy—animal	1
	map04146	Peroxisome	3
	map04145	Phagosome	1
	map04144	Endocytosis	3
	map04140	Autophagy—animal	1
Cell growth and death	map04110	Cell cycle	1
	map04114	Oocyte meiosis	1
	map04210	Apoptosis	1
	map04217	Necroptosis	3
	map04215	Apoptosis—multiple species	1
	map04214	Apoptosis—fly	1
Organismal Systems			
Endocrine system	map04935	Growth hormone synthesis, secretion, and action	1
	map04910	Insulin signaling pathway	2
	map04912	GnRH signaling pathway	1
	map04913	Ovarian steroidogenesis	1
	map04914	Progesterone-mediated oocyte maturation	1
	map04916	Melanogenesis	1
	map04917	Prolactin signaling pathway	1
	map04926	Relaxin signaling pathway	2
	map04921	Oxytocin signaling pathway	1
	map04920	Adipocytokine signaling pathway	1
	map04923	Regulation of lipolysis in adipocytes	1
	map04925	Aldosterone synthesis and secretion	1
	map04924	Renin secretion	1
	map03320	PPAR signaling pathway	2
Sensory system	map04750	Inflammatory mediator regulation of TRP channels	2
	map04740	Olfactory transduction	1
Nervous system	map04728	Dopaminergic synapse	1
	map04726	Serotonergic synapse	1
	map04730	Long-term depression	1
	map04723	Retrograde endocannabinoid signaling	1
	map04722	Neurotrophin signaling pathway	1
Immune system	map04657	IL-17 signaling pathway	1
	map04659	Th17 cell differentiation	1
	map04658	Th1 and Th2 cell differentiation	1

	map04624	Toll and Imd signaling pathway	3
	map04664	Fc epsilon RI signaling pathway	1
	map04660	T cell receptor signaling pathway	1
	map04620	Toll-like receptor signaling pathway	2
	map04625	C-type lectin receptor signaling pathway	1
	map04622	RIG-I-like receptor signaling pathway	1
	map04621	NOD-like receptor signaling pathway	3
Circulatory system	map04260	Cardiac muscle contraction	2
	map04261	Adrenergic signaling in cardiomyocytes	1
	map04270	Vascular smooth muscle contraction	1
Digestive system	map04978	Mineral absorption	1
	map04972	Pancreatic secretion	4
	map04970	Salivary secretion	2
	map04977	Vitamin digestion and absorption	1
	map04974	Protein digestion and absorption	2
	map04975	Fat digestion and absorption	3
Environmental adaptation	map04713	Circadian entrainment	1
	map04714	Thermogenesis	2
Excretory system	map04961	Endocrine and other factor-regulated calcium reabsorption	1
Development and regeneration	map04361	Axon regeneration	1
	map04380	Osteoclast differentiation	1
Aging	map04213	Longevity regulating pathway—multiple species	2
	map04212	Longevity regulating pathway—worm	4
Human Diseases			
Cancer: overview	map05231	Choline metabolism in cancer	1
	map05235	PD-L1 expression and PD-1 checkpoint pathway in cancer	1
	map05203	Viral carcinogenesis	4
	map05205	Proteoglycans in cancer	1
	map05202	Transcriptional misregulation in cancer	4
	map05200	Pathways in cancer	2
Cancer: specific types	map05225	Hepatocellular carcinoma	1
	map05226	Gastric cancer	1
	map05212	Pancreatic cancer	1
	map05217	Basal cell carcinoma	1
	map05210	Colorectal cancer	1
	map05224	Breast cancer	1
Cardiovascular disease	map05418	Fluid shear stress and atherosclerosis	1
	map05414	Dilated cardiomyopathy (DCM)	1
	map05410	Hypertrophic cardiomyopathy (HCM)	1

	map05412	Arrhythmogenic right ventricular cardiomyopathy (ARVC)	1
Drug resistance: antineoplastic	map01522	Endocrine resistance	1
Endocrine and metabolic disease	map04934	Cushing syndrome	1
	map04932	Non-alcoholic fatty liver disease (NAFLD)	2
	map04933	AGE-RAGE signaling pathway in diabetic complications	1
	map04930	Type II diabetes mellitus	1
	map04931	Insulin resistance	1
Immune disease	map05322	Systemic lupus erythematosus	7
Infectious disease: bacterial	map05110	Vibrio cholerae infection	1
	map05120	Epithelial cell signaling in Helicobacter pylori infection	1
	map05135	Yersinia infection	2
	map05134	Legionellosis	1
	map05131	Shigellosis	6
	map05130	Pathogenic Escherichia coli infection	2
	map05133	Pertussis	2
	map05132	Salmonella infection	2
	map05152	Tuberculosis	2
Infectious disease: parasitic	map05140	Leishmaniasis	1
	map05142	Chagas disease (American trypanosomiasis)	2
	map05143	African trypanosomiasis	1
	map05145	Toxoplasmosis	2
	map05144	Malaria	1
Infectious disease: viral	map05166	Human T-cell leukemia virus 1 infection	3
	map05167	Kaposi sarcoma-associated herpesvirus infection	1
	map05164	Influenza A	1
	map05165	Human papillomavirus infection	2
	map05162	Measles	2
	map05161	Hepatitis B	2
	map05168	Herpes simplex virus 1 infection	2
	map05169	Epstein-Barr virus infection	2
	map05170	Human immunodeficiency virus 1 infection	2
Neurodegenerative disease	map05020	Prion diseases	1
	map05012	Parkinson disease	1
	map05010	Alzheimer disease	4
	map05016	Huntington disease	4
	map05017	Spinocerebellar ataxia	2
	map05014	Amyotrophic lateral sclerosis (ALS)	2
Substance dependence	map05034	Alcoholism	7

Table S3. Analysis of KEGG annotation of co-expressed DEGs in three comparations.

Category	Pathway ID	Description	Gene Numbers
Metabolism			
	map00510	N-Glycan biosynthesis	1
	map00511	Other glycan degradation	1
Glycan biosynthesis and metabolism	map00513	Various types of N-glycan biosynthesis	1
	map00533	Glycosaminoglycan biosynthesis—keratan sulfate	1
Genetic Information Processing			
Replication and repair	map03420	Nucleotide excision repair	1
Organismal System			
Immune system	map04624	Toll and Imd signaling pathway	1
Cellular Processes			
Cell growth and death	map04115	p53 signaling pathway	1
Transport and catabolism	map04142	Lysosome	1
Human disease			
	map05203	Viral carcinogenesis	1
Cancer: overview	map05202	Transcriptional misregulation in cancer	2
Immune disease	map05322	Systemic lupus erythematosus	1
Infectious disease: bacterial	map05131	Shigellosis	1
Substance dependence	map05034	Alcoholism	1

Table S4. Detailed information about immune genes that were annotated in RNA-seq.

	Gene Accession	Description
PRRs (Pathogen recognition receptors)		
PGRP-LB	XM_022335679.1	Peptidoglycan-recognition protein LB-like
PGRP-LF	XM_022339197.1	Peptidoglycan-recognition protein LF-like
B-1,3-GBP	XM_022352399.1	Beta-1,3-glucan-binding protein-like
B-1,3-GBP1	XM_022338890.1	Beta-1,3-glucan-binding protein 1-like
C-type lectin 37Db	XM_022331369.1	C-type lectin 37Db-like
C-type lectin B	XM_022333733.1	C-type lectin domain family 12 member B-like
Hemocytin	XM_022333596.1	Hemocytin
Galectin-3	XM_022332636.1	Galectin-3-like
Galectin-4	XM_022328656.1	Galectin-4-like
Galectin-8	XM_022351104.1	Galectin-8-like
Dscam2	XM_022344067.1	Down syndrome cell adhesion molecule-like protein Dscam2
Draper	XM_022344313.1	Protein draper
Toll pathway		
Toll	XM_022343147.1	Protein toll-like
Toll3	XM_022350549.1	Toll-like receptor 3
Toll6	XM_022335164.1	Toll-like receptor 6
Toll7	XM_022343764.1	Toll-like receptor 7
Toll-T	XM_022338051.1	Toll-like receptor Tollo

TIP	XM_022333701.1	Toll-interacting protein-like
Myd88	XM_022332200.1	Myeloid differentiation primary response protein MyD88-like, transcript variant X1
IRAK4(Tube)	XM_022352033.1	Interleukin-1 receptor-associated kinase 4-like
Pelle	XM_022329577.1	Probable serine/threonine-protein kinase PBL3
Dorsal	XM_022339686.1	Embryonic polarity protein dorsal-like
Defensin	XM_022330678.1	Defensin-like
IMD pathway (Immune deficiency pathway)		
UbcE2	XM_022332363.1	Ubiquitin-conjugating enzyme E2 N
Tak1	XM_022333544.1	TGF-beta-activated kinase 1 and MAP3K7-binding protein 1-like
IKK	XM_022333943.1	Inhibitor of nuclear factor kappa-B kinase subunit alpha
IAP-1	XM_022336615.1	Death-associated inhibitor of apoptosis 1-like
Ankyrin3	XM_022337253.1	Ankyrin-3-like
JAK-STAT pathway (Janus kinase/ signal transducers and activators of transcription pathway)		
JAK	XM_022343450.1	Janus kinase and microtubule-interacting protein 2-like
Hopscotch	XM_022343232.1	Tyrosine-protein kinase hopscotch, transcript variant X1
STAT-5B	XM_022335891.1	Signal transducer and activator of transcription 5B, transcript variant X1
SCS5	XM_022350557.1	Suppressor of cytokine signaling 5-like
Serine protease cascade and PPO cascade (Prophenoloxidase cascade)		
SP7	XM_022335287.1	Serine protease 7-like
SP29	XM_022349553.1	Serine protease 29-like, transcript variant X1
SP38	XM_022344796.1	Serine protease 38-like
SP44	XM_022349564.1	Serine protease 44-like
SP48	XM_022349830.1	Serine protease 48-like
SP55	XM_022351379.1	Serine protease 55-like
Venom	XM_022333724.1	Venom protease-like
SP-gd	XM_022345077.1	Serine protease gd-like
SP-nudel	XM_022351685.1	Serine protease nudel-like
SP-persephone	XM_022349134.1	Serine protease persephone-like
SP-snake	XM_022345106.1	Serine protease snake-like
SP-stubble	XM_022339670.1	Serine proteinase stubble
SPI(Serpin)	XM_022339915.1	Serine protease inhibitor-like
K-SPI-B6	XM_022328190.1	Kunitz-type serine protease inhibitor B6-like
SPIB5	XM_022338506.1	Serpin B5
SPIB10	XM_022341426.1	Serpin B10-like, transcript variant X1
SPIB8	XM_022343420.1	Serpin B8-like
SPI88Ea	XM_022344286.1	Serine protease inhibitor 88Ea-like
PPAF	XM_022338213.1	Tryptase-like
PO2(PPO)	XM_022339577.1	Phenoloxidase 2-like
MP1	XM_022331231.1	Melanization protease 1-like
Proclotting	XM_022328195.1	Proclotting enzyme-like
Lysozyme	XM_022329786.1	Lysozyme-like
Lysozyme2	XM_022329791.1	Lysozyme 2-like
LysozymeC1	XM_022333781.1	Lysozyme C-1-like
Lysozyme3	XM_022334309.1	Lysozyme 3-like
I-Lysozyme3	XM_022351808.1	Invertebrate-type lysozyme 3-like

JNK pathway (c-Jun N-terminal kinase pathway)		
MAPK1	XM_022328174.1	Mitogen-activated protein kinase 1
MAPK7	XM_022342543.1	Mitogen-activated protein kinase kinase kinase 7-like
TNF-α	XM_022349170.1	Lipopolysaccharide-induced tumor necrosis factor-alpha factor-like
TNF-α-H	XM_022342028.1	Lipopolysaccharide-induced tumor necrosis factor-alpha factor homolog
JNK	XM_022349131.1	Stress-activated protein kinase JNK-like
JNK-IP1	XM_022330773.1	JNK-interacting protein 1
Hsp68	XM_022341134.1	Heat shock protein 68-like

Table S5. Primers used in transcriptomic data validation.

Primer	Primer Sequence (5′-3′)	Gene Accession
q-Def- XM_022330678.2-F	TACTGCTGTTCTTCTGCGGGTT	XM_022330678.2
q-Def- XM_022330678.2-R	GGTCGCTACAGCCGATTCACG	
q-SPK12-XM_022329132.1-F	GCATGATCTCGACTGTGGCT	XM_022329132.1
q- SPK12-XM_022329132.1-R	TCGTCCTCATCACTATAACC	
q-SPK12-2-XM_022348572.1-F	TGGCATCACAGAAGACAAT	XM_022348572.1
q- SPK12-2-XM_022348572.1-R	CTCTGGTGGATCGCTATC	
q-TFAP-XM_022341733.1-F	CCGTCTCACCAATCAACA	XM_022341733.1
q- TFAP-XM_022341733.1-R	GTCGTCTGCACTTGGAAG	
q-Per-XM_022350387.1-F	CACTCCTACACAACTGACA	XM_022350387.1
q-Per-XM_022350387.1-R	TGGCACTCTTGGATTCATAA	
q-Nrflp6-XM_022331081.1-F	GCATCTCCTACTTCCATCAG	XM_022331081.1
q- Nrflp6-XM_022331081.1-R	TTCACACCGCCTCCATAT	
q-NADH-XM_022340437.1-F	TGTCACCAGCAAGAGTATG	XM_022340437.1
q-NADH-XM_022340437.1-R	CTCGCATCAAGTCAAGAAC	
q-Rib-XM_022331424.1-F	TGTTGAACGAGCCTGATG	XM_022331424.1
q- Rib-XM_022331424.1-R	GGTGGTCAAGTCCATTCC	
q-Trf-XM_022336705.1-F	TCTATGGATGCTGGCTACT	XM_022336705.1
q-Trf-XM_022336705.1-R	GGCGTTGGATGACTTCTT	
q-SAP-XM_022331325.1-F	ATCACTCTCGCATTGGAAT	XM_022331325.1
q-SAP-XM_022331325.1-R	CGGACACAAGTTATCTACAC	
q-AFP-XM_022339714.1-F	CTTCATTCTCGCTCTGTCT	XM_022339714.1
q-AFP-XM_022339714.1-R	TGCCAGTATCCATGCTTG	
q-CP-XM_022333780.1-F	CTCTGGTGGATCGCTATC	XM_022333780.1
q- CP-XM_022333780.1-R	AGCGTTGAATCCGTTGAC	
q-NRFP96-XM_022338165.1-F	TGCGATTCCTCAGTCTTG	XM_022338165.1
q-NRFP96-XM_022338165.1-R	AATGTCCTCAGCGTCTTG	
q-P450-XM_022338406.1-F	CGCCAAGAATGTTCAGTTAT	XM_022338406.1
q- P450-XM_022338406.1-R	CTTCGCACCATCAGTCAT	
q-SPK12H4-XM_022344846.1-F	GTGAGAAGGCTGTCATAGA	XM_022344846.1
q-SPK12H4-XM_022344846.1-R	CCATAGAATCTGTGCTCCA	
q-β-GBP-XM_022349166.1-F	CAACCGACCTTATTAGATGAC	XM_022349166.1
q-β-GBP-XM_022349166.1-R	GACTGGAGGAAGTATTGAGAA	
q-T-β-H-XM_022328872.1-F	GGAACTCAAAGAACTCAAC	XM_022328872.1
q- T-β-H-XM_022328872.1-R	ACTTCGGCAACAATATGGT	
q-Pce-XM_022350883.1-F	AGGCTCAGGTGGATATAAGA	XM_022350883.1
q- Pce-XM_022350883.1-R	ATCCTCCAGCACATATTACTC	
q-Lrdd-XM_022350638.1-F	TTCCTCCATTCGCACAAT	XM_022350638.1
q-Lrdd-XM_022350638.1-R	ATCGTTAGAAGCAAGACTCA	

q-Actin-F<br>q-Actin-R	TGGACTTCGAGCAGGAAATGG<br>ACGTCGCACTTCATGATC	XM_022345417.2

Table S6. Relative expression of selected four genes in RNA-seq and RT-qPCR.

Gene	Group	RNA-seq	RT-qPCR	Consistency
	SF/CK	1.01	1.368	yes
Toll	SV/CK	1.101	0.674	
	CI/CK	1.075	1.090	yes
	SF/CK	0.38	1.349	
Myd88	SV/CK	0.01	0.724	yes
	CI/CK	1.197	1.006	yes
	SF/CK	1.013	1.482	yes
PPO	SV/CK	0.823	0.881	yes
	CI/CK	0.753	0.698	yes
	SF/CK	7.024	1.407	yes
TNF-α	SV/CK	1.637	0.512	
	CI/CK	0.284	0.926	yes

RRSV-free BPH were treated with Tween-80 (CK) or *Metarhizium anisopliae* YTTR (SF) and RRSV-carrying BPH were also treated with Tween-80 (SV) or *M. anisopliae* YTTR (CI).
